# Experimental Model of Proximal Junctional Fracture after Multilevel Posterior Spinal Instrumentation

**DOI:** 10.1155/2016/8058796

**Published:** 2016-08-17

**Authors:** Jean-Marc Mac-Thiong, Annie Levasseur, Stefan Parent, Yvan Petit

**Affiliations:** ^1^Hôpital du Sacré-Coeur de Montreal, 5400 Boulevard Gouin Ouest, Montreal, QC, Canada H4J 1C5; ^2^Department of Surgery, Université de Montreal, CP 6128, Succursale Centre-Ville, Montreal, QC, Canada H3C 3J7; ^3^Department of Surgery, CHU Sainte-Justine, 3175 Cote-Sainte-Catherine, Montreal, QC, Canada H3T 1C5; ^4^Department of Mechanical Engineering, Ecole de Technologie Supérieure, 1100 Notre-Dame, Montreal, QC, Canada H3C 1K3

## Abstract

There is a high risk of proximal junctional fractures (PJF) with multilevel spinal instrumentation, especially in the osteoporotic spine. This problem is associated with significant morbidity and possibly the need for reoperation. Various techniques have been proposed in an attempt to decrease the risk of PJF but there is no experimental model described for* in vitro* production of PJF after multilevel instrumentation. The objective of this study is to develop an experimental model of PJF after multilevel posterior instrumentation. Initially, four porcine specimens including 4 vertebrae and instrumented at the 3 caudal vertebrae using a pedicle screw construct were subjected to different loading conditions. Loading conditions on porcine specimens involving cyclic loading along the axis of the center vertebral body line, with constrained flexion between 0° and 15° proximally, and fully constraining the specimen distally resulted in a fracture pattern most representative of a PJF seen clinically in humans, so to undergo human cadaveric testing with similar loading conditions was decided. Clinically relevant PJF were produced in all 3 human specimens. The experimental model described in this study will allow the evaluation of different parameters influencing the incidence and prevention of PJF after multilevel posterior spinal instrumentation.

## 1. Introduction

With the advent of stronger spinal fixation techniques, there is increased risk of proximal junctional fractures (PJF) with multilevel surgeries, especially in the osteoporotic spine [1, 2]. Two types of PJF typically occur, either fracture of the supra-adjacent uninstrumented vertebra or fracture of the upper instrumented vertebra [2]. PJF can be associated with significant morbidity and possibly the need for reoperation to extend the construct proximally [3, 4]. In an attempt to decrease the risk of PJF, different techniques have been suggested, such as prophylactic cement augmentation [5, 6] and the use of wires [7] or hooks [8] at the top of the construct. Unfortunately, there is no consensus on an experimental model to use for* in vitro* production of PJF after multilevel spinal instrumentation, which makes it difficult to compare different methods to prevent PJF. The objective of the current study is therefore to develop an experimental model of PJF after multilevel posterior spinal instrumentation.

## 2. Materials and Methods

This research has been approved by the Institutional Review Board. All fresh-frozen specimens were stored at −20°C before being thawed at room temperature for 24 hours in order to harvest and instrument the spine. The spines were then frozen and stored at −20°C, before being thawed at room temperature for 24 hours to perform the biomechanical testing. There were therefore 2 freeze-thaw cycles involved for all specimens. Considering that the mechanical properties of the spine can change with multiple freeze-thaw cycles [9–11], all tests were performed within the same day after the second freeze-thaw cycle for all specimens.

### 2.1. Porcine Cadaveric Testing

Initially, four immature porcine specimens aged 3 months consisting in a segment of 4 mid-thoracic vertebrae were obtained. Care was taken to preserve the intervertebral discs, articular facet capsules, and posterior ligamentous complex. The 3 caudal vertebrae for each specimen were instrumented posteriorly using 4.0 mm diameter multiaxial titanium pedicle screws and 5.5 mm diameter titanium rods (Expedium, DePuy Spine Inc., Raynham, MA, USA), while leaving the cephalad vertebra uninstrumented. The proximal and distal halves of corresponding cephalad and caudal vertebrae were embedded into polyester resin (Bondo Autobody Filler, Bondo Corporation, Atlanta, GA, USA) in order to have a flat surface to apply the load and to standardize the positioning during testing. The specimens were installed in a servohydraulic testing machine (858 Mini Bionix II, MTS Systems Corporation, Eden Prairie, MN, USA) after mounting the most caudal vertebra in a customized aluminum frame.

**Figure 1:**
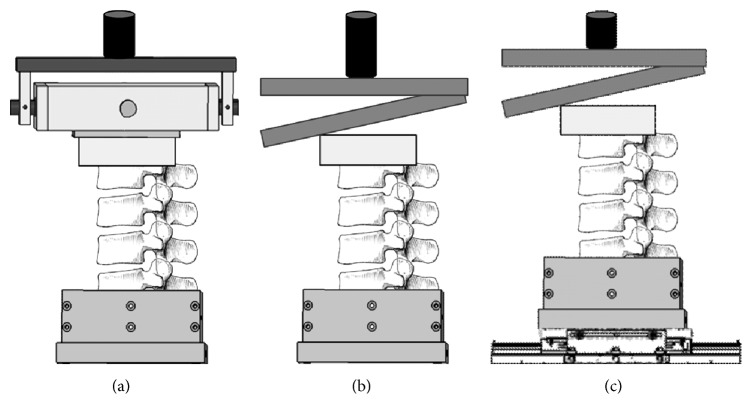
Experimental setup used for compression loading of porcine spinal segments. (a) Specimens 1 and 2 were unconstrained in flexion and extension proximally, while being fully constrained distally. (b) Specimen 3 was constrained in flexion proximally (between 0° and 15°), while being fully constrained distally. (c) Specimen 4 was constrained in flexion proximally (between 0° and 15°), while being unconstrained distally for anterior-posterior translation.

Table 1 describes the loading conditions for the porcine specimens. The loading mode refers to the application of continuous or cyclic loading. The loading control describes the method by which compression loading was controlled by the testing machine. Loading control by displacement was associated with continuous loading at 25 mm/min, while loading control by force was associated with cyclic loading at 1 Hz. In case of cyclic loading, the high-end of the loading interval was increased after a specific number of cycles, while the low-end remained constant. The loading axis refers to the alignment of the actuator with respect to the specimen before the application of compression loading. Boundary conditions determine the constraints for the upper and lower ends of the specimen. Proximally, unconstrained flexion-extension or constrained flexion between 0° and 15° was used. Distally, the specimens were either fixed (fully constrained) or unconstrained for anterior-posterior translation (Figure 1).

After testing, the instrumentation was removed and visual inspection was performed to identify the presence of any fracture or disruption of the specimens. The loading conditions associated with a fracture that was most similar to a PJF seen clinically on humans were retained to define the loading conditions for the second part of the study on human specimens. Clinically relevant PJF was defined as a fracture of the supra-adjacent uninstrumented vertebra and/or fracture of the upper instrumented vertebra [2].

### 2.2. Human Cadaveric Testing

**Figure 2:**
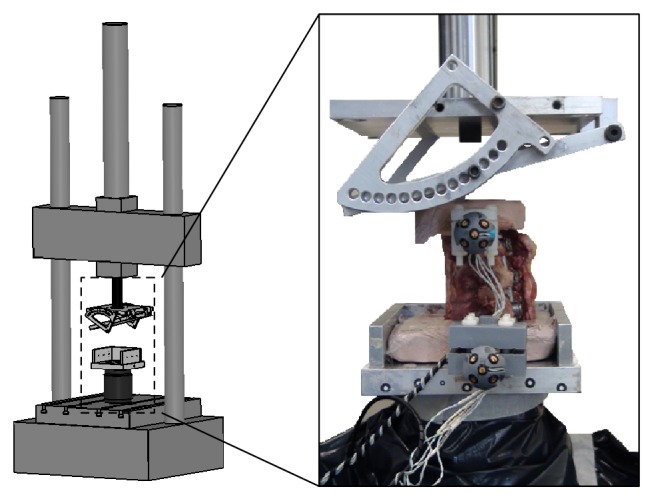
Experimental setup used for compression loading of human spinal segments. A schematic illustration of the setup placed into the servohydraulic testing machine is shown on the left, while a picture of the fixture and human specimen construct is shown on the right.

Three human cadaveric specimens consisting in a segment of 4 vertebrae (T2–T5) were used. They were aged 61 (specimen A), 73 (specimen B), and 65 years (specimen C) at death. Care was taken to preserve the intervertebral discs, articular facet capsules, and posterior ligamentous complex. CT scan with calibration phantoms was obtained initially in order to rule out the presence of preexisting fractures and to estimate the apparent bone density. The 3 distal vertebrae were instrumented posteriorly using 5.0 mm diameter multiaxial titanium pedicle screws and 5.5 mm diameter titanium rods, while leaving the proximal vertebra uninstrumented. The specimens were installed on the testing machine after fixing the distal half of the lower vertebra (fully constrained) in a customized aluminum frame using polyester resin (Figure 2). The proximal half of the supra-adjacent uninstrumented vertebra was embedded into polyester resin with a flat surface to apply the load. Compressive loading was achieved using an aluminum plate with an inclination of 15° fixed to the actuator aligned with the center vertebral body line, allowing constrained flexion between 0° and 15° proximally.

Each specimen was submitted to cyclic loading between 50 N and 1000 N at 1 Hz. The low-end value of 50 N was set to reflect a compressive load from a head weighing approximately 5 kg. The high-end value was estimated from the linear regression equation suggested by Lindsey et al. [12] to predict ultimate compressive force from bone mineral density (BMD) for single human thoracic vertebrae. As recommended for cyclic testing [12], the high-end value of 1000 N corresponds to approximately 80% of the ultimate compressive force for the average BMD of 0.262 g/cc observed for the three specimens.

Axial force and displacement were recorded at 25 Hz using a linear variable displacement transducer and a 15 kN load cell, respectively. The presence of a PJF was detected by the first discontinuity (sudden increase) in the displacement range reflecting crack formation and loss of stability [13]. After testing, specimens were examined using CT scan followed by visual inspection.

## 3. Results

### 3.1. Porcine Cadaveric Testing

**Figure 3:**
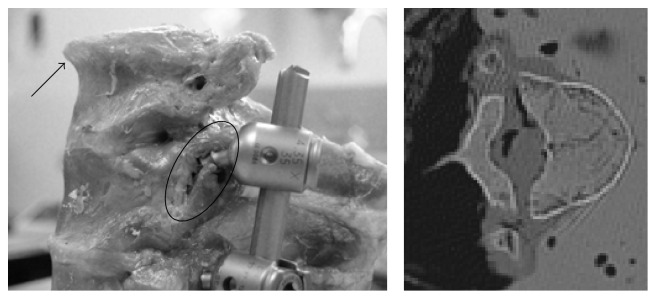
Specimen 3 after loading. Visual inspection revealed an upper endplate fracture of the supra-adjacent uninstrumented vertebra (arrow). Fracture of the upper instrumented vertebra extending to the left pedicle was also visible (oval). An axial CT scan cut of the supra-adjacent uninstrumented vertebra shows the pattern of the upper endplate fracture.

A fracture was obtained for each specimen. Loading of specimen 1 resulted in a compression fracture of the supra-adjacent uninstrumented vertebra and a flexion distraction injury of the upper instrumented vertebra. Specimen 2 was associated with compression fractures of the upper and middle instrumented vertebrae. Compression fractures occurred through the supra-adjacent uninstrumented and upper instrumented vertebrae in specimen 3 (Figure 3). A flexion distraction injury through the lower instrumented vertebra was observed in specimen 4. Since the fracture pattern associated with specimen 3 was most representative of a PJF seen clinically, to undergo human cadaveric testing with loading conditions similar to those used for specimen 3 was decided, that is, constrained cyclic compression with a proximal inclination of 15°.

### 3.2. Human Cadaveric Testing

**Figure 4:**
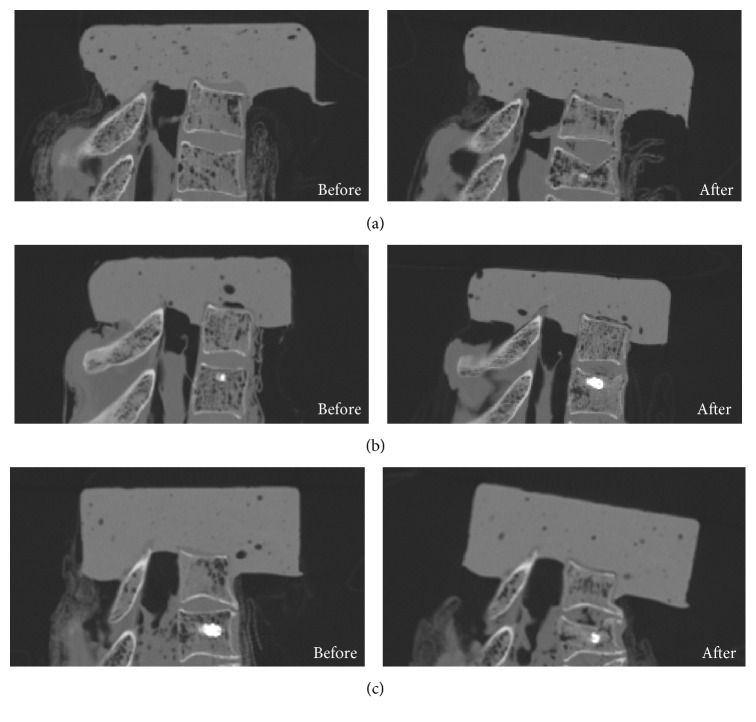
CT scan images showing proximal junctional fractures of supra-adjacent uninstrumented and upper instrumented vertebrae after mechanical testing for specimens A, B, and C.

**Figure 5:**
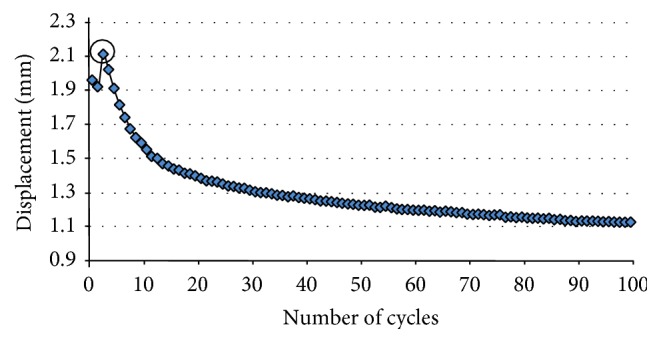
Graph showing the measured displacement with respect to the number of cycles for specimen A, depicting the moment when the fracture occurs (circled) with a sudden increase in displacement followed by a gradual decrease.

Compression fractures of both supra-adjacent uninstrumented (T2) and upper instrumented (T3) vertebrae were obtained for all three specimens (Figure 4), after 3, 11, and 3 cycles, respectively, for specimens A, B, and C.  Figure 5 shows the typical pattern for the displacement-cycles curve until fracture.

## 4. Discussion

The current study proposes an experimental model of PJF after multilevel posterior spinal instrumentation. By defining the biomechanical conditions required to reliably obtain a PJF experimentally, it will be possible to assess the impact of various parameters on the incidence and prevention of PJF. The proposed model will be invaluable to testing of new strategies in order to prevent the risk of PJF, which represents a major challenge in spinal surgery [14]. The porcine cadaveric testing was specifically useful for comparing different loading conditions in order to produce the PJF on human specimens. Porcine specimens are readily available and inexpensive and therefore could be used to explore multiple testing conditions or other types of lesions or surgical constructs. However, considering the anatomical differences between porcine and human spines, results from porcine spines should be validated using human cadaveric spines, as was done in the current study. This is particularly true when using immature porcine specimens, since PJF typically occur in older patients with osteoporosis [1, 2]. This study has shown that the experimental setup using human cadaveric spines is adequate for future evaluation of PJF.

The first part of the study on porcine specimens allowed determining the loading conditions required to produce PJF after multilevel posterior spinal instrumentation. Unfortunately, there is no recognized experimental model described in the literature for* in vitro* production of PJF after multilevel spinal instrumentation. When searching through the literature pertaining to the* in vitro* production of fractures on uninstrumented porcine spines, most studies have used continuous displacement control to obtain a fracture [15–17]. However, there is no consensus regarding the optimal rate of continuous displacement to use, and the rate therefore varies significantly between studies, although it has an important influence on the resulting fracture morphology [18]. As for cyclic loading on porcine vertebrae, there is no clear recommendation in the literature on the optimal conditions for producing fractures. In our study, we wanted to include a set of experiments with continuous displacement control because it is the loading mode mostly used in previous studies. We also wanted to include cyclic loading in order to account for physiological activities of daily living where the upper body weight is applied cyclically to the spine. Considering the absence of consensus for loading conditions, the authors selected their parameters in order to comply with ASTM F 1717 “Standard Test Methods for Spinal Implant Constructs in a Vertebrectomy Model” recommending values up to 25 mm/min and 5 Hz, respectively, for static and fatigue testing of spinal implant constructs. Cyclic loading was set at 1 Hz to relate with a stride rate typically found in older individuals [19] who are at higher risk for PJF.

The second part of the study on human specimens confirmed the relevance of constrained cyclic compression loading to produce PJF. One important benefit from cyclic and force-controlled loading is that it better reproduces the cyclic loads typical for humans during daily activities. Clinically, patients with PJF usually do not report a specific traumatic event, and PJF are only noted from routine radiographic follow-up. The low-end value of the loading interval was set at 50 N in order to simulate the presence of constant gravitational loads from the weight of the head. Accordingly, to modify the low-end value based on the weight of the body segment above the spinal levels to be tested is recommended. As for the high-end value of the loading interval, a load of 1000 N was set based on an existing equation relating BMD to ultimate compressive load for thoracic vertebrae [12]. Since only a small number of cycles were required to obtain a PJF, improvements in the prevention of PJF provided by alternative techniques could be difficult to detect under these loading conditions. Therefore, a load equivalent to 70% of the estimated ultimate compressive force, as opposed to 80% in the present study, would be preferable in future studies involving the same vertebral levels and similar BMD. Indeed, the smaller number of cycles to failure in the current study compared to the data from Lindsey et al. [12] supports the fact that the instrumented spine is at increased risk for a fracture proximally. It is however difficult to adjust the testing protocol to a single clinical scenario because even if PJF most often occur within the first 8 months following the surgery, there is a wide variability as they can be observed within the first months or even years after surgery [1, 2, 20, 21].

As for the loading axis, it was kept at the center of the vertebral body line because of the increased probability of producing a flexion distraction injury when positioned more anteriorly, as seen in porcine specimen 1. This behavior is similar to that seen clinically in humans for whom flexion distraction injuries are associated with a center of rotation anterior to the spine. Similarly, leaving the upper or lower end of the specimen unconstrained in flexion-extension or anterior-posterior translation can lead to an increased likelihood of obtaining a flexion distraction injury, as seen in porcine specimens 1 and 4. Accordingly, there was no evidence of flexion distraction injury in any of the human specimens when aligning the loading axis along the center vertebral body line and fully constraining the segments proximally and distally. Finally, the proximal flexion for the impact was set at 15° in order to impose slight local kyphosis between the supra-adjacent uninstrumented and upper instrumented vertebrae. It is believed that local kyphosis will add a slight flexion component to the compression loading, thereby increasing the likelihood of obtaining a compression fracture as seen typically in PJF, rather than a burst fracture often associated with pure axial compression loading.

Incremental loading was not used in the human cadaveric testing although it was used successfully in porcine specimens. It can be difficult to directly compare multiple prevention techniques if different increments for loading have been reached. On the opposite, keeping a single loading interval for all tests will allow direct comparisons of the number of cycles required for a PJF.

Unfortunately, the experimental model does not take into account the potential influence of muscles in the occurrence of PJF, and it is recognized as a limitation for this study. However, when using the current model to compare different techniques for preventing PJF that do not involve a procedure targeting the muscles, it is less likely that neglecting the effect of muscles would significantly influence the comparison. In addition, while we have only applied axial forces with minor variations in the loading axis, we did not consider other forces that can also contribute to PJF. Indeed, it is believed that human spines are subjected to multiple different forces, axial and eccentric loads, and twisting moments, all of which contribute to the development of PJF [22]. Finally, the authors recommend performing all biomechanical testing within the same day after the same freeze-thaw cycle in order to take into account the effect of multiple freeze-thaw cycles on the mechanical properties of the spine [9–11].

## Figures and Tables

**Table 1:** Loading conditions for all four porcine specimens.

Loading characteristics	Specimen 1	Specimen 2	Specimen 3	Specimen 4
Loading control	Displacement	Force	Force	Displacement

Loading mode	Continuous	Cyclic at 1 Hz	Cyclic at 1 Hz	Continuous

Loading speed and amplitude	25 mm/min	Incremental after every 1000 cycles at 50–200 N, 50–400 N, 50–600 N, 50–800 N, 50–1000 N	Incremental after 150 cycles at 200–800 N, 100 cycles at 200–1300 N, 400 cycles at 200–2500 N, 100 cycles at 200–3500 N	25 mm/min

Loading axis	Anterior vertebral body line	Center vertebral body line	Center vertebral body line	Center vertebral body line

Boundary conditions	Upper: free flexion-extension Lower: fixed	Upper: free flexion-extension Lower: fixed	Upper: 0°–15° constrained flexion Lower: fixed	Upper: 0°–15° constrained flexion Lower: free anterior-posterior
